# SMG6’s PIN (PilT N-Terminus) Domain Is Required for Nonsense-Mediated mRNA Decay (NMD) *In Vivo*

**DOI:** 10.3390/cells15030282

**Published:** 2026-02-02

**Authors:** Baihui Chai, Xiao Tan, Yan Li, Chengyan Chen, Xin Ma, Tangliang Li

**Affiliations:** 1 State Key Laboratory of Microbial Technology, Shandong University, Qingdao Campus, Qingdao 266237, China; 2 Zhejiang Key Laboratory of Medical Epigenetics, School of Basic Medical Sciences, Hangzhou Normal University, Hangzhou 311121, China; 3 College of Animal Science and Technology, Qingdao Agricultural University, Qingdao 266109, China

**Keywords:** SMG6, PIN domain, embryonic stem cell, differentiation, tissue homeostasis

## Abstract

Nonsense-mediated mRNA decay (NMD) is a highly conserved RNA quality and quantity surveillance machinery in eukaryotic cells, serving as an important node in the post-transcriptional gene expression. Previous studies using the complete knockout of individual NMD factors in cells or animals reveal that NMD deficiency causes developmental defects and compromises tissue homeostasis. However, because most NMD factors participate in multiple molecular functions, a direct link between NMD and cell fate determination is missing. SMG6 is a core NMD effector and the only endoribonuclease among all NMD factors. The NMD function of SMG6 is exclusively mediated by its PIN (PilT N-terminus) domain. In this study, we engineered a mouse model with the capability of specifically deactivating the SMG6’s PIN domain/endoribonuclease activity (*Smg6*-PIN^F/F^), but not knocking out the complete SMG6 protein. We found that SMG6’s PIN domain is essential for NMD activity in embryonic stem cells (ESCs) and various tissues of adult mice. Furthermore, loss of SMG6’s PIN domain is dispensable for the mouse ESC self-renewal, but severely compromises the differentiation, which consequently causes the mutant mice to die during the process of organogenesis. Through the induced deletion of SMG6’s PIN domain in adult mice, we found that loss of SMG6’s NMD function affects the homeostasis of several mouse tissues, including the testis and the intestine. In sum, our study establishes a mechanistic link between NMD per se and cell fate determination of mouse ESCs, as well as in the tissues of adult mice, where cell fate transitions are actively ongoing. The *Smg6*-PIN^F/F^ mouse line could be a valuable strain for elucidating the biology of NMD per se.

## 1. Introduction

The functionality of cells and maintenance of organismal homeostasis are determined by the faithful transmission of genetic information from DNA to proteins. Pre-messenger RNAs (pre-mRNAs) are transcribed from gene-coding DNA sequences and further processed into mature mRNAs, which are translated into functional proteins to build cellular structure and establish cellular function. However, due to genetic mutations, errors in DNA transcription, and aberrant pre-mRNA processing (including splicing errors and alternative splicing), premature termination codons can be introduced into mRNAs, compromising the faithful transmission of genetic information [1,2,3]. Nonsense-mediated RNA decay is a well-conserved RNA surveillance mechanism that was initially identified for degrading mRNAs containing premature termination codons. Thus, NMD plays essential roles in safeguarding the transcriptome quality and ensuring the faithful transfer of DNA information to proteins. In recent years, NMD has also been found to be responsible for destabilizing a specific group of PTC-free RNA transcripts with features of long 3′UTR, uORFs, etc. [4,5,6]. Thus, NMD, up to date, is considered as an RNA quality and quantity regulatory mechanism, degrading 3–10% of the transcriptome of eukaryotic cells [3,6], and acts as a mechanism of post-transcriptional gene expression regulation [1].

The NMD machinery is very conservative in eukaryotic species [7]. Up to now, in mammalian species, at least 17 proteins have been identified to actively participate in the steps of NMD target recognition, degradation initiation and execution [8]. Three up-frameshift proteins (UPF1, UPF2, and UPF3B), together with four suppressors with morphogenetic effect on genitalia (SMG1, SMG5, SMG6, and SMG7), compose the core NMD machinery [3,7]. Studies using animal models showed that these NMD factors safeguard the differentiation of embryonic stem cells [9,10,11,12,13], neural stem cells [14,15], spermatogonia [16,17,18], craniofacial neural crest cells [19], and oligodendrocyte precursors [20], indicating that NMD is a licensing mechanism in the cell fate transition of different types of stem cells [6]. Accordingly, *Upf1*, *Upf2*, *Smg1*, *Smg5*, and *Smg6* were essential for zebrafish embryos’ development [21]. Null mutation of NMD factors *Smg1*, *Smg5*, *Smg6*, *Upf1*, *Upf2* or *Upf3a* in mice leads to embryonic lethality. However, whether the lethality of these NMD factor knockouts is exclusively due to the deficiency of NMD per se is not conclusive, which is mainly due to the fact that the majority of NMD factors function beyond degrading the NMD targets with features of PTC, long 3′UTR, uORF, etc. NMD factors have diverse molecular functions in genomic stability maintenance (SMG1, UPF1, UPF2, and SMG7), cell cycle regulation and cell death (EJC components, UPF2, UPF1, and SMG6), telomere maintenance (SMG1, UPF1, UPF2, SMG5, SMG6, and SMG7), and ubiquitin modification and protein degradation (UPF1) [22]. Thus, complete knockout of an NMD factor in human cell lines or animal models is useful, but not sufficient, to establish a direct link between NMD per se and its biological functions.

SMG6, a homolog of Est1, a telomerase activator in the budding yeast, is a key NMD effector in the RNA degradation step of NMD pathway. The mammalian SMG6 protein is composed of several domains: the N-terminal of SMG6, including two conserved EBM1 and EBM2 motifs which are thought to interact with the exon-junction complex (EJC), is mainly related to the telomerase activity; the C-terminal of SMG6 includes the 14-3-3-like domain and the PIN domain [23]. The 14-3-3-like domain of SMG6 can mediate the interaction with phosphorylated UPF1, a prerequisite of NMD target-degradation initiation. The phosphorylated UPF1 interacts with the 14-3-3-like domain of SMG6, and recruits SMG6 to the vicinity of PTCs of NMD targets [7]. With the PIN domain at the very C-Terminal of SMG6, SMG6 executed endoribonuclease function [24,25,26]. By far, SMG6 is the only endoribonuclease in the NMD pathway. We previously showed that the complete knockout of SMG6 is dispensable for the self-renewal of mouse embryonic stem cells. SMG6 null mice die between E7.5–12.5 due to an essential role of SMG6 in ESC differentiation [10]. This finding was further confirmed by Huth et al. [12]. Furthermore, by ectopically expressing different SMG6-truncated proteins in SMG6 null ESCs, we found that the expression of a truncated SMG6 protein without its PIN domain does not rescue the differentiation defects of SMG6 null ESCs, revealing that SMG6’s PIN domain is vital for NMD and differentiation of ESC in vitro. Whether SMG6’s PIN domain is required for embryogenesis *in vivo* is largely unknown.

In this study, through the classical gene-targeting strategy conducted in mESCs, we generated a novel NMD mouse model with the capability to specifically knock out the SMG6’s PIN domain (*Smg6*-PIN^F/F^). With this specific mouse line, we have investigated the biological roles of SMG6’s endoribonuclease activity and found that NMD per se is required for the mESC differentiation and early embryonic development. Furthermore, our pilot study revealed that NMD per se is essential for homeostasis across multiple tissues in adult mice. Thus, the Smg6-PIN^F/F^ mouse is a reliable model line to delineate the functions of NMD per se.

## 2. Materials and Methods

### 2.1. Generation of SMG6-PIN Domain Conditional Knockout Mouse Line (Smg6-PIN^F/F^)

**Figure 1:**
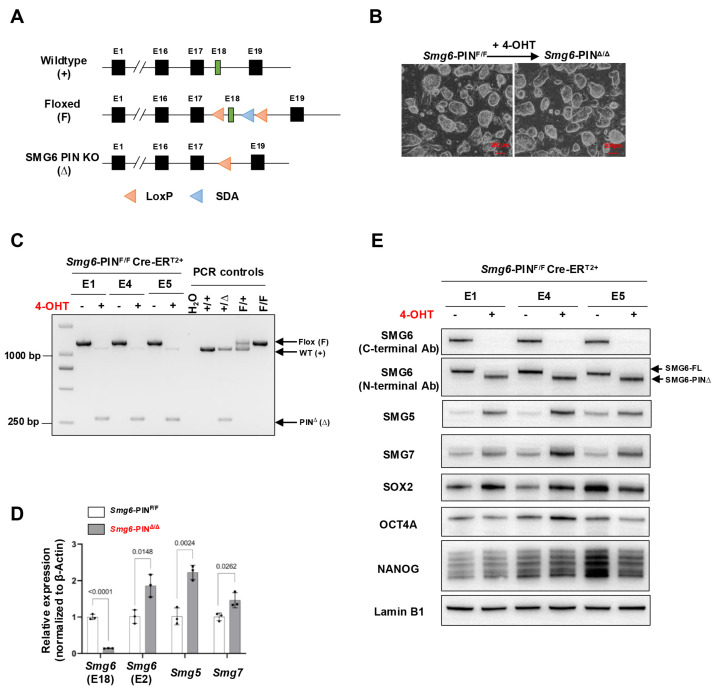
Characterization of SMG6 PIN domain conventional knockout ESCs. (**A**) Schematic diagram of different alleles of the SMG6’s PIN domain conditional knockout mouse (SDA: self-deletion anchor site). (**B**) Representative images of control (*Smg6*-PIN^F/F^ Cre-ER^T2+^; shortly *Smg6*-PIN^F/F^) and SMG6’s PIN domain knockout (*Smg6*-PIN^Δ/Δ^ Cre-ER^T2+^; shortly *Smg6*-PIN^Δ/Δ^) ESCs. Note: The 4-OHT is used to induce the *Smg6* exon 18 deletion in *Smg6*-PIN^F/F^ Cre-ER^T2+^ ESCs. (**C**) PCR analysis on the deletion of *Smg6* exon 18 in *Smg6*-PIN^F/F^ Cre-ER^T2+^ after 5 days of 4-OHT induction. (**D**) qPCR analysis to detect the expression of gene transcripts for *Smg6*, *Smg5* and *Smg7*. Note: Two sets of qPCRs are used to detect the expressions of *Smg6* E18 and E2, respectively. (**E**) WB analysis on the expression of full-length SMG6 (SMG6-FL), mutated SMG6 (SMG6-PIN∆), SMG5, SMG7, SOX2, OCT4A and NANOG in *Smg6*-PIN^F/F^ Cre-ER^T2+^, and 4-OHT-treated *Smg6*-PIN^F/F^ Cre-ER^T2+^ ESCs. Expression of Lamin B1 in each sample was used as the loading control. Note: The E1, E4, and E5, genotyped as *Smg6*-PIN^F/F^ Cre-ER^T2+^, are three mESC lines derived from 3 independent E3.5 blastocytes. The E1, E4, and E5 ESC lines were used for (**B**–**E**). Unpaired Student’s *t*-test was carried out for statistical analysis.

The generation of SMG6 PIN domain conditional knockout mouse line was conducted with a commercial gene-targeting service with Cyagen Biosciences Inc. (Suzhou, China). In the mouse, the endoribonuclease domain of SMG6 (the PIN domain) is encoded by exons 16–19 of *Smg6* gene. Thus, we designed to conditionally delete exon 18 of *Smg6*, thereby removing the very C-terminus of SMG6 including two of the three key aspartates (D1352 and D1391) in the catalytic endoribonuclease domain (Figure 1A, and Supplementary Figure S1).

To conditionally delete the *Smg6* exon 18 in the ES cells, the targeting vector was designed with a 4.4 kb 5′ arm and a 2.4 kb 3′ arm and generated using BAC clones from the C57BL/6J RPCI-23 BAC library. The 5′ loxP DNA sequence, the target for the Cre-recombinase, was inserted into intron 17. The second loxP site, together with a self-excision Neo cassette flanked by Rox sites that can be cleaved by the testes-specific Dre recombinase, was inserted into intron 18. The negative selection marker diphtheria toxin A (DTA) cassette was placed upstream of the 5′ homology arm. The linearized targeting vector was introduced into Cyagen’s proprietary TurboKnockout ES cells (on a C57Bl/6N background) by electroporation. Homologous recombinant clones were isolated using the positive (neomycin) and negative (DTA) selection procedures. Southern blotting analysis was conducted with restriction enzyme-digested genomic DNAs from ESC clones to detect homologous recombination events. The integration of the 5′ homologous arm was performed using BstEII restriction enzyme and Neo probe, which gave 10.22 kb (targeted allele) fragments. Southern hybridization to verify the successful integration of 3′ homologous arm was performed by digestion with BsoBI restriction enzyme, and the Neo probe produced 6.70 kb (targeted allele) fragments. The targeted ES cells were introduced into host embryos, and 4–8 microinjected cell embryos were then surgically transferred into pseudo-pregnant (surrogate) mothers to generate F0 heterozygous floxed mice (*Smg6*-PIN^F/+^) on a C57BL/6N background.

To genotype different alleles of *Smg6*-PIN conditional knockout mouse, two primers (*Smg6*-F, 5′-TTAGAAATGTTCCTGAGCTGGGTAGT-3′; *Smg6*-R, 5′-CCCACCTCCAGTTCCAGTTAGCCT-3′) were used: wild-type allele (WT) 1061 bps, floxed allele 1222 bps, and PIN domain knockout allele (Δ) 260 bps.

### 2.2. Generation of the SMG6 PIN Domain Conventional Knockout Mouse Line

To generate the conventional knockout allele of SMG6 PIN domain (Δ), the *Smg6*-PIN^F/+^ mouse was crossed with the Nestin-Cre transgenic mouse line. Crossing *Smg6*-PIN^F/+^ Nestin-Cre^+^ males with C57BL/6 wild-type females will produce offsprings with the genotype of *Smg6*-PIN^Δ/+^ due to leaky expression of Cre-recombinase in the testis of *Smg6*-PIN^F/+^Nestin-Cre^+^ males [27] (Supplementary Figure S2). To investigate the survival of *Smg6*-PIN^Δ/Δ^ embryos, *Smg6*-PIN^Δ/+^ males and females were used for plug-checking. The embryos at different stages were isolated and genotyped.

### 2.3. Generation of the Inducible SMG6 PIN Domain Knockout Mouse and Embryonic Stem Cell Lines

*Smg6*-PIN^F/+^ mice were bred with CreER^T2+^ transgenic mice to generate the inducible SMG6 PIN domain knockout mouse line (*Smg6*-PIN^F/F^; CreER^T2+^) (Supplementary Figure S2).

SMG6-PIN domain-inducible knockout ESCs (*Smg6*-PIN^F/F^; CreER^T2+^) were generated from E3.5 blastocysts from the crossing between *Smg6*-PIN^F/F^; CreER^T2+^ males and Smg6-PIN^F/F^ females by following a previously established protocol [28]. Three individual ESC lines derived from different embryos (E1, E4, and E5; all genotyped as *Smg6*-PIN^F/F^ CreER^T2+^) were generated. The ESC lines were maintained in the ESC culture medium: High glucose DMEM (Gibco (Grand Island, NY, USA), 11995-065) supplemented with 15% FCS (Gibco, 10099141), 1 × Pen/Strep (Gibco, 15140122), 1 × GlutaMAX^TM^ (Gibco, 25050061), 1 × Sodium pyruvate (Gibco, 11360070), 1 × Non-essential amino acids (Millipore (Burlington, MA, USA), TMS-001-C), 1 μM 2-metacptoethanol (Gibco, 21985032), and 1000 units/ml LIF (Millipore, ESG1107). ESC lines were passaged every 3 days. To delete SMG6’s PIN domain, *Smg6*-PIN^F/F^; CreER^T2+^ ESCs were treated with 4-OHT (Sigma-Aldrich (Burlington, MA, USA), 1 μM) for 5 days.

To delete the SMG6’s PIN domain in adult *Smg6*-PIN*^F/F^*; CreER^T2+^ mice, Tamoxifen (Sigma-Aldrich, 15 mg/mL diluted in corn oil) was delivered into 8-week-old mice by intraperitoneal injection for three consecutive days (75 mg/kg body weight) [28].

### 2.4. Spontaneous Differentiation Assay of mESCs

The spontaneous differentiation assay was conducted following a method described previously [13]. In brief, 2 × 10^5^ *Smg6*-PIN^Δ/Δ^ ESCs and their parental ESC lines were cultured on gelatin-coated 6-well dishes using the ESC medium without LIF for 5 days. The culture medium was changed every 2 days. After fixation with 4% PFA for 5 min at room temperature, the differentiated ES cells were stained for 30 min at 37 °C using an AP staining kit (Beyotime Biotechnology (Shanghai, China), C3250S).

### 2.5. Histological Analysis

Mouse tissues were collected at the indicated timepoints, fixed with 4% paraformaldehyde (PFA) at 4 °C overnight, dehydrated in ethanol, embedded in paraffin, and finally cut into 5 μm thick sections. Hematoxylin and eosin (HE) staining was performed with a commercial HE-staining kit (E607318, Sangon Biotech (Shanghai, China)). Histological images were scanned with Olympus SlideView VS200 microscopy system and analyzed with the cellSens software (V4.2, Olympus (Hamburg, Germany)).

### 2.6. qRT–PCR Assay

Total RNAs from ESCs or mouse tissue samples were isolated using Trizol following the company’s manual. The cDNA was synthesized using the HiScript^®^ III RT SuperMix for qPCR (R323, Vazyme (Nanjing, China)) kit. qRT-PCR was performed in triplicate using 2 × TSINGKE^®^ Master qPCR Mix (SYBR Green I). β-Actin was used as a reference control, and qRT-PCR data were analyzed using the ΔΔCq method. The primers used in this study are listed in Table 1 and Table 2.

### 2.7. Immunoblotting Analysis

Total ESC proteins were extracted by lysis using RIPA buffer containing 1mM PMSF, and protein expression levels were analyzed using SDS-PAGE as well as enhanced chemiluminescence detection solutions. The primary antibodies used were as follows: Rabbit anti-SMG6 (C-terminal) (1:1000, Abcam (Cambridge, UK), ab87539), Rabbit anti-SMG6 (N-terminal) (1:1000, sigma, HPA042932), Rabbit anti-SMG5 (1:1000, Abcam, ab33033), Rabbit anti-SMG7 (1:1000, sigma, SAB2701517), Mouse anti-Lamin B1 (1:3000, Santa Cruz Biotechnology (Dallas, TX, USA), sc-374015), Rabbit anti-eIF4A2 (1:1000, Abcam, ab31218), Rabbit anti-ATF4 (1:1000, Abcam, ab184909), Rabbit anti-DDIT3 (1:1000, Proteintech (Rosemont, IL, USA), 15204-1-AP), Mouse anti-β-Actin (1:3000, Sigma-Aldrich, A5441), Rabbit anti-c-MYC (1:1000, Cell Signaling Technology, (Danvers, MA, USA), 5605S), Rabbit anti-SOX2 (1:1000, Cell Signaling Technology, 23064s), Rabbit anti-OCT4A (1:1000, Cell Signaling Technology 2840S), and Rabbit anti-NANOG (1:1000, Cell Signaling Technology, 8822S).

### 2.8. Statistical Analysis

The unpaired Student’s *t*-test was used for statistical analysis. Statistical analysis was performed using GraphPad Prism 5.0 and was considered statistically significant when the *p*-value < 0.05.

## 3. Results

### 3.1. Generation and Characterization of Smg6-PIN Inducible Knockout and Conventional Knockout (Smg6-PIN^Δ/Δ^) ESC Line

The *Smg6*-PIN^F/+^ mice were bred with CreER^T2+^ transgenic mice and finally generated the *Smg6*-PIN^F/F^ CreER^T2+^ mouse line (Supplementary Figure S2). We isolated E3.5 *Smg6*-PIN^F/F^ CreER^T2+^ blastocysts and established three *Smg6*-PIN^F/F^ CreER^T2+^ ESC lines (ESC lines E1, E4, and E5). We added 4-Hydroxytamoxifen (4-OHT) to these *Smg6*-PIN^F/F^ CreER^T2+^ ESC lines to induce the deletion of Exon 18 of *Smg6*. PCR, qRT-PCR, and Western blot confirmed the deletion efficiency of *Smg6* exon 18 and PIN domain of SMG6. PCR analysis revealed complete removal of *Smg6* exon 18 in 4-OHT-treated E1, E4, and E5 ESC lines (Figure 1C). qPCR analysis using the primers specifically detecting *Smg6* exon 18 further revealed that mRNA transcripts from 4-OHT-treated E1, E4, and E5 ESC lines do not have *Smg6* exon 18. Intriguingly, qPCR primers specific for exon 2 of *Smg6* revealed that mutated *Smg6* mRNA transcripts without exon 18 are efficiently produced in 4-OHT-treated E1, E4, and E5 ESC lines (Figure 1D). Lastly, we used two commercial SMG6 antibodies to confirm the expression of the truncated SMG6 protein without its PIN domain. Immunoblotting with an SMG6 C-terminal antibody showed that protein samples from 4-OHT-treated E1, E4, and E5 ESC lines lack immunoreactivity (Figure 1E). However, the use of SMG6’s N-terminal antibody revealed that truncated SMG6 proteins were expressed in 4-OHT-treated E1, E4, and E5 ESC lines (Figure 1E).

Taken together, these results indicate that we have successfully generated stably passaged *Smg6*-PIN^Δ/Δ^ ESC lines. The *Smg6*-PIN^F/F^ mouse line could be used to precisely delineate the SMG6’s NMD function *in vivo*.

### 3.2. NMD Is Inhibited in Smg6-PIN^Δ/Δ^ ESCs

**Figure 2:**
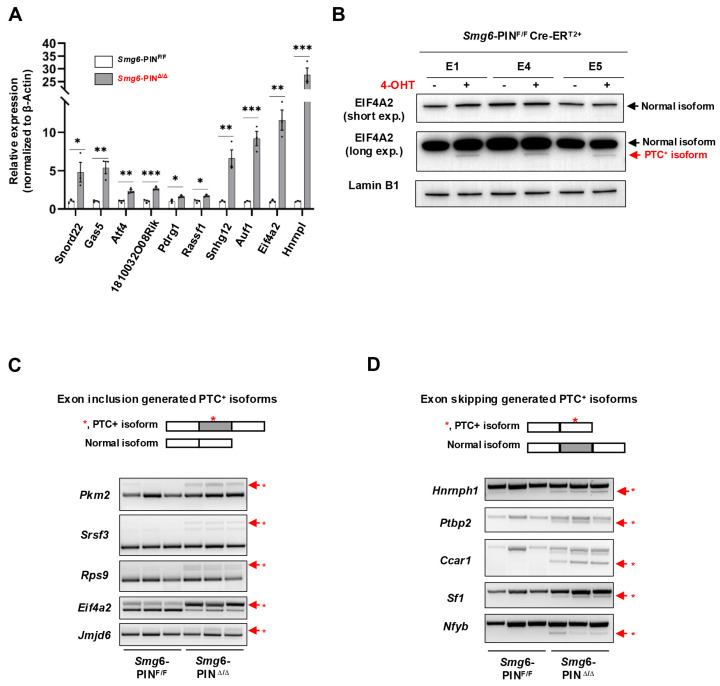
SMG6’s PIN domain is required for NMD in mESC. (**A**) qPCR analysis on the expressions of classical NMD target gene transcripts in control (*Smg6*-PIN^F/F^) and *Smg6*-PIN^Δ/Δ^ ESCs. (**B**) WB analysis on the protein expression of full-length and PTC^+^ isoforms of EIF4A2 in control ESCs and *Smg6*-PIN^Δ/Δ^ ESCs. Note: The 4-OHT is used to induce the *Smg6* exon 18 deletion in *Smg6*-PIN^F/F^ Cre-ER^T2+^ ESCs to generate *Smg6*-PIN^Δ/Δ^ ESCs. (**C**,**D**) RT-PCR analysis to show the accumulation of PTC^+^ transcripts produced by the exon-inclusion and exon-skipping events of alternative splicing. Red asterisks denote the PTC^+^ isoforms. Note: The E1, E4 and E5, genotyped as *Smg6*-PIN^F/F^ Cre-ER^T2+^, are three mESC lines derived from 3 independent E3.5 blastocytes. The E1, E4, and E5 ESC lines were used for Figure 2. Unpaired Student’s *t*-test was carried out for statistical analysis. *, *p* < 0.05; **, *p* < 0.01; ***, *p* < 0.001.

Next, we determined whether NMD was attenuated in *Smg6*-PIN^Δ/Δ^ embryonic stem cells. mRNA transcripts of NMD factors are NMD targets [29]. qRT–PCR and Western blot analyses confirmed that as compared with their parental control ESC lines, *Smg6*-PIN^Δ/Δ^ ESCs expressed higher levels of Smg5 and Smg7 in mRNA and protein (Figure 1D,E). To further investigate NMD inhibition in *Smg6*-PIN^Δ/Δ^ ESCs, we then analyzed the expression level of endogenous NMD targets, including PTC-containing transcripts, transcripts with long or intron-containing in the 3′ untranslated region, and transcripts with uORFs in the 5′ untranslated region [4]. qPCR analysis revealed that the expression levels of the classical NMD targets, including *Snord22*, *Gas5*, *Atf4*, *Eif4a2*, *1810032O08Rik*, *Pdrg1*, *Rassf1*, *Auf1*, and *Hnrnpl*, were all significantly elevated in *Smg6*-PIN^Δ/Δ^ ESCs [12,30] (Figure 2A). *Snord22* and *Gas5* are well-described snoRNA host genes containing introns in their 3′UTRs [30]. The activating transcription factor 4 (ATF4) plays a key role in physiological responses to various stresses [31]. uORFs were found in the 5′ untranslated region of *Atf4* mRNA. Our results showed that *Atf4* mRNA levels were significantly increased (Figure 2A). eIF4A2 is a subunit of the eIF4F complex involved in 5′ cap recognition and mRNA binding to the ribosome [32]. Huth et al. showed that *eIF4A2* encodes two splicing isoforms: a full-length *eIF4A2* and a predicted PTC-containing *eIF4A2* isoform (*eIF4A2^PTC^*) [12]. Our results showed a significant increase in Eif4a2 PTC^+^ mRNA and the production of eIF4A2-truncated protein in *Smg6*-PIN^Δ/Δ^ ESCs (Figure 2A,B).

The alternative splicing (AS) of pre-mRNAs is a conserved mechanism in generating transcriptome complexity. Around 40% of AS events produce mRNA containing premature termination codons (PTCs) [33]. Alternative splicing-coupled NMD (AS-NMD) plays a vital role in regulating gene expression [34,35]. When NMD is inhibited, PTC-containing mRNA transcripts will accumulate in cells. Here, we analyzed the expression of gene-specific PTC isoforms generated by exon-inclusion events (*Pkm2*, *Rps9*, *Srsf3*, *Eif4a2*, *Jmjd6*) and exon-skipping events (*Hnrnph1*, *Ptbp2*, *Ccar1*, *Nfyb*, Sf1) and found that all these PTC^+^ isoforms are enriched in *Smg6*-PIN^Δ/Δ^ ESCs (Figure 2C,D).

In conclusion, SMG6’s PIN domain is essential for NMD in ESC.

### 3.3. The Smg6-PIN^Δ/Δ^ Mouse Is Embryonic Lethal

**Figure 3:**
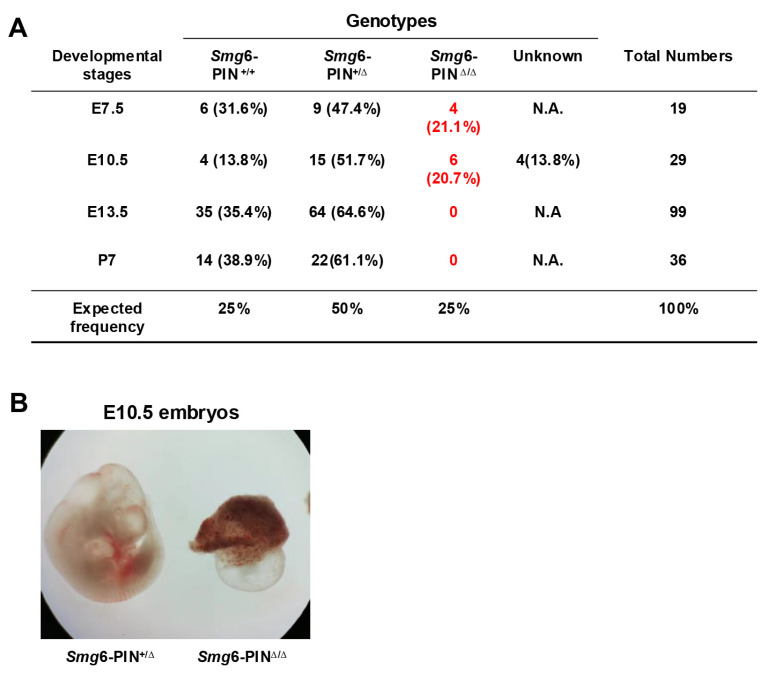
*Smg6*-PIN^Δ/Δ^ mouse is embryonic lethal. (**A**) Mendelian analysis on the mice and embryos from the intercrossing of *Smg6*-PIN^+/Δ^ mice. (**B**) Representative images of *Smg6*-PIN^+/Δ^ and *Smg6*-PIN^Δ/Δ^ embryos at E10.5. Note: At E10.5, the *Smg6*-PIN^Δ/Δ^ embryo is severely retarded.

Loss of complete SMG6 protein in mice causes embryonic lethality, and SMG6 null embryos die between E7.5–12.5 [10]. To investigate whether deletion of SMG6‘s PIN domain is compatible with life, we first generated SMG6 PIN domain conventional knockout allele (*Smg6*-PIN^Δ^) by crossing the *Smg6*-PIN domain conditional knockout mouse (*Smg6*-PIN^F/F^) with the Nestin-Cre transgenic line (Supplementary Figure S2). Intercrossing of *Smg6*-PIN^Δ/+^ mice produces no viable *Smg6*-PIN^Δ/Δ^ mice on postnatal day 7 (P7) (Figure 3A). Furthermore, no *Smg6*-PIN^Δ/Δ^ embryo could be found at E13.5 (Figure 3A). Intriguingly, *Smg6*-PIN^Δ/Δ^ have no visible defects as compared with control littermates at E7.5 (Figure 3A), while, at E10.5, only severely underdeveloped *Smg6*-PIN^Δ/Δ^ embryos can be found in the uterus of pregnant mothers (Figure 3B). Thus, SMG6’s PIN domain is essential for the embryonic development. *Smg6*-PIN^Δ/Δ^, like the Smg6 complete knockout mouse (*Smg6*^Δ/Δ^), dies after E7.5 when the mouse organogenesis starts.

These data indicate that NMD per se is required for the mouse organogenesis.

### 3.4. Smg6-PIN^Δ/Δ^ ESCs Have Differentiation Defects

**Figure 4:**
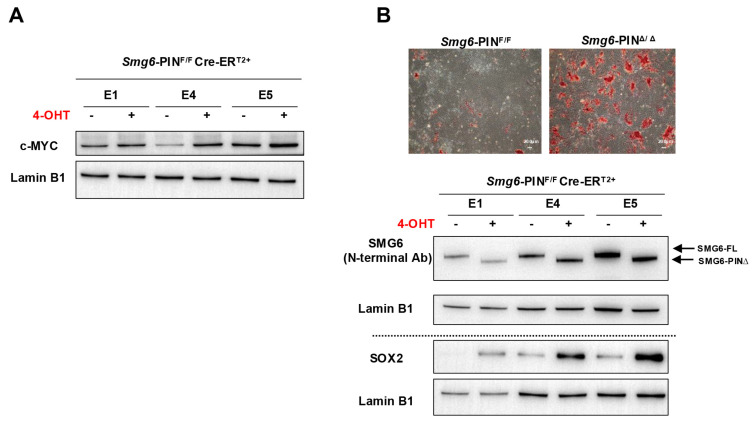
The self-renewal and differentiation of *Smg6*-PIN^Δ/Δ^ ESC. (**A**) Western blot analysis on the expression of pluripotency factor c-MYC in *Smg6*-PIN^F/F^ and *Smg6*-PIN^Δ/Δ^ ESCs. (**B**) Spontaneous differentiation of *Smg6*-PIN^F/F^ and *Smg6*-PIN^Δ/Δ^ ESCs. Upper panel: representative images of AP-stained differentiation cultures from *Smg6*-PIN^F/F^ and *Smg6*-PIN^Δ/Δ^ ESCs at day 5. Lower panel: protein expression of SMG6 and SOX2 in *Smg6*-PIN^F/F^ and *Smg6*-PIN^Δ/Δ^ differentiation cultures (Day 5). Expression of Lamin B1 in each sample was used as the loading control. Note: The E1, E4, and E5, genotyped as *Smg6*-PIN^F/F^ Cre-ER^T2+^, are three mESC lines derived from 3 independent E3.5 blastocytes. The 4-OHT is used to induce the *Smg6* exon 18 deletion and thus to generate *Smg6*-PIN^Δ/Δ^ ESCs.

Embryonic stem cells can proliferate in an undifferentiated state or be induced to differentiate into various cell types, providing a powerful model for studying early mammalian development. Our group and Huth et al. previously showed that knocking out SMG6, SMG5, or SMG7 is dispensable for ESC self-renewal but results in failure of mESC differentiation [10,12,13]. Thus, we investigate whether *Smg6*-PIN^Δ/Δ^ ESC have defects in self-renewal and differentiation. In ESC culture conditions, *Smg6*-PIN^Δ/Δ^ ESCs show indistinguishable morphology as compared with their parental ESC clones (Figure 1B). Western blot analysis further revealed that protein expressions of several core pluripotency factors (OCT4, SOX2, and NANOG) were comparable between *Smg6*-PIN^Δ/Δ^ ESCs and their parental ESC clones (Figure 1E). We previously demonstrated that under feeder-layer-supported ESC culture condition, c-MYC protein levels were significantly increased in SMG6 complete knockout ESCs [10]. In *Smg6*-PIN^Δ/Δ^ ESCs, the protein expression of c-MYC was significantly increased, as shown with Western blot analysis (Figure 4A).

To test the differentiation capacity of *Smg6*-PIN^Δ/Δ^ ESCs, we performed ESC spontaneous differentiation assay. AP staining of ESC differentiation culture on day 5 showed a higher alkaline phosphatase activity in the *Smg6*-PIN^Δ/Δ^ ESC differentiation culture as compared with the control group (Figure 4B). Western blot analysis further confirmed that *Smg6*-PIN^Δ/Δ^ ESCs had high levels of SOX2, a stem cell marker (Figure 4B).

These findings indicate that NMD per se is required for the proper differentiation of ESCs.

### 3.5. SMG6’s PIN Domain Is Required for NMD in Adult Mice

**Figure 5:**
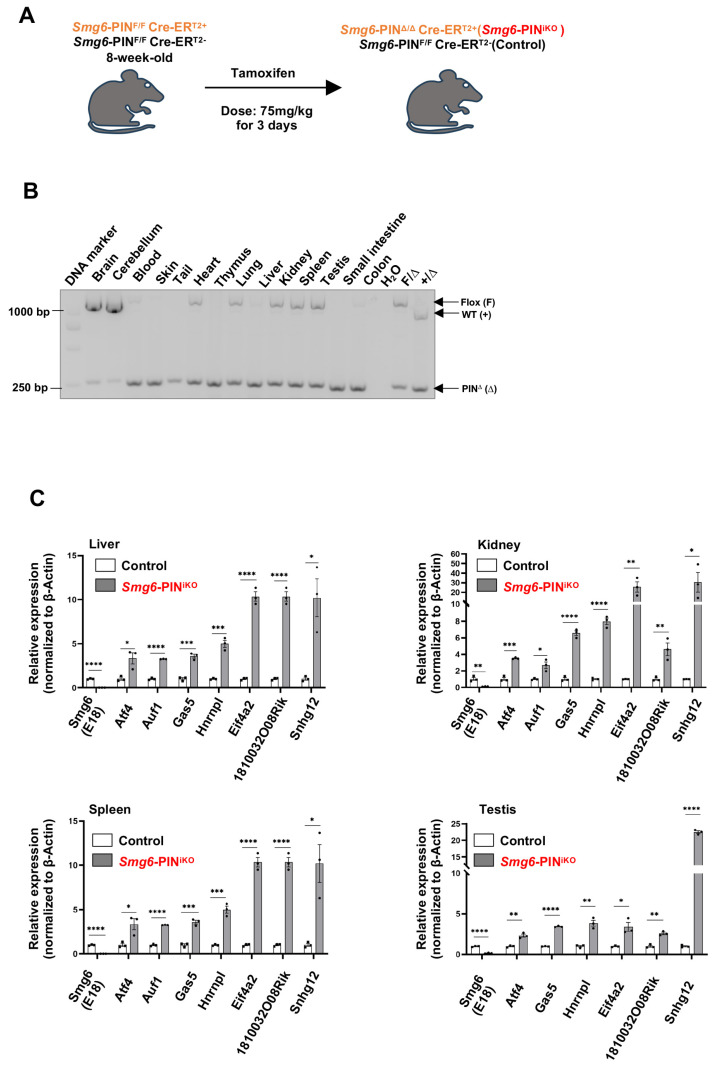
Deletion of SMG6’s PIN domain causes NMD inhibition in adult mice. (**A**) Experimental scheme to delete SMG6’s PIN domain in mice. (**B**) PCR analysis on the excision efficiency of *Smg6* exon 18 in Tamoxifen-treated *Smg6*-PIN^F/F^ Cre-ER^T2+^ mice (one month after the last Tamoxifen treatment; denoted as *Smg6*-PIN^iKO^). (**C**) qPCR analysis on the expressions of classical NMD target gene transcripts in control (*Smg6*-PIN^F/F^ Cre-ER^T2−^ mice treated with Tamoxifen) and *Smg6*-PIN^iKO^ mice (n = 3 for each group). Note: Unpaired Student’s *t*-test was carried out for statistical analysis. *, *p* < 0.05; **, *p* < 0.01; ***, *p* < 0.001; ****, *p* < 0.0001.

To study the biological roles of NMD per se in the tissue maintenance of adult mice, two-month-old male control mice (genotyped as *Smg6*-PIN^F/F^ CreER^T2−^) and SMG6-PIN domain-inducible knockout mice (genotyped as *Smg6*-PIN^F/F^ CreER^T2+^) were treated with Tamoxifen (Figure 5A). The deletion efficiency was confirmed with PCR and qPCR assays (Figure 5B,C). PCR analysis on tissues isolated from *Smg6*-PIN^F/F^ CreER^T2+^ (one month after the last injection of Tamoxifen) revealed almost complete deletion of *Smg6* exon 18 in tissues, including blood, skin, thymus, liver, small intestine, and colon. Thus, we named the Tamoxifen-treated *Smg6*-PIN^F/F^ CreER^T2+^ mice as *Smg6*-PIN^iKO^. Of note, Tamoxifen treatment of *Smg6*-PIN^F/F^ CreER^T2+^ does not trigger efficient SMG6’s PIN domain deletion in brain regions (Figure 5B). qPCR analysis on liver, kidney, spleen, and testis tissues from *Smg6*-PIN^iKO^ mice further confirmed that *Smg6* exon 18 is efficiently deleted (Figure 5C).

To further investigate whether SMG6’s PIN domain functions in NMD in adult tissue, we quantified the expression levels of various NMD target mRNAs by qPCR. Consistent with the studies in ESCs, the expression levels of NMD target mRNAs, including *Atf4*, *Auf1*, *Gas5*, *Hnrnpl*, *Eif4a2*, *1810032O08Rik* and *Snhg12*, were significantly increased in the liver, kidney, spleen, and testis of *Smg6*-PIN^iKO^ mice (one month after the last injection of Tamoxifen) (Figure 5C). Furthermore, we used RT-PCR to analyze the expression level of gene-specific PTC isoforms generated by exon-inclusion events (*Rps9*, *Eif4a2*, *Jmjd6*) and exon-exclusion events (*Ccar1*, *Nfyb*, *Sf1*) in the liver, kidney, spleen, and testis of control and *Smg6*-PIN^iKO^ mice. All these gene-specific PTC isoforms are enriched upon SMG6’s PIN domain deletion (Supplementary Figure S3). All these data indicate that SMG6’s PIN domain is required for NMD *in vivo*.

### 3.6. Loss of NMD Per Se Affects Homeostasis of Multiple Tissues in Adult Mice

**Figure 6:**
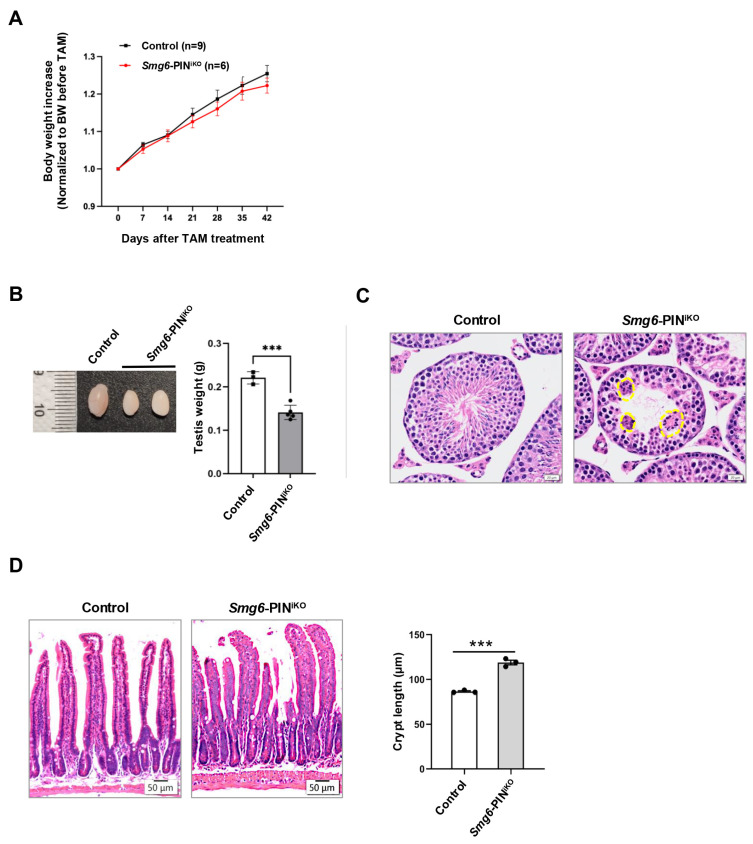
Compromised tissue homeostasis in *Smg6*-PIN^iKO^ mice. (**A**) Body weight growth of Tamoxifen-treated *Smg6*-PIN^F/F^ Cre-ER^T2−^ (Control) and *Smg6*-PIN^F/F^ Cre-ER^T2+^ (*Smg6*-PIN^iKO^) mice. Note: Tamoxifen treatment of *Smg6*-PIN^F/F^ Cre-ER^T2−^ does not cause deletion of *Smg6* exon 18, serving as the control group; while Tamoxifen treatment of *Smg6*-PIN^F/F^ Cre-ER ^T2+^ mice will delete *Smg6* exon 18, producing *Smg6*-PIN^iKO^ mice. (**B**,**C**) Testis atrophy in *Smg6*-PIN^iKO^ mice. The testis weight and representative HE images of seminiferous tubules from control and *Smg6*-PIN^iKO^ mice are shown (3 controls and 5 *Smg6*-PIN^iKO^ mice were used). Note: The yellow circles mark the multinucleated giant cells. (**D**) Enlargements of crypts in *Smg6*-PIN^iKO^ mice. The representative HE images of the intestinal epithelium from control and *Smg6*-PIN^iKO^ mice and the quantification of crypt length are shown (3 controls and 3 *Smg6*-PIN^iKO^ mice were used). Note: (**B**–**D**) are summarized from mice one month after the last Tamoxifen injection. Unpaired Student’s *t*-test was carried out for statistical analysis. ***, *p* < 0.001.

Most of the NMD factors are essential for embryonic development [6,13,36,37], which hinders the analysis of the effects of NMD deficiency in tissue maintenance of adult animals. In our study, efficient deletion of SMG6’s PIN domain or abolishment of SMG6’s NMD activity does not cause acute lethality in mice. Except for a slightly reduced body weight, SMG6’s PIN domain-deleted mice (*Smg6*-PIN^iKO^) have no gross morphological defect as compared with controls in 6 weeks after the last Tamoxifen injection (Figure 6A). However, at one month after Tamoxifen injection, testis weight was significantly reduced in *Smg6*-PIN^iKO^ mice (Figure 6B). Histochemical analysis revealed that the *Smg6*-PIN^iKO^ testes exhibited significant reductions in elongating spermatids and the appearance of multinucleated giant cells, indicating a defect in spermatogenesis (Figure 6C). This finding supported a previous study on the essential roles of SMG6 in spermatogenesis [38].

In the intestinal tissue of *Smg6*-PIN^iKO^ mice, we noticed that the crypts, including the intestinal stem cells and transient amplifying progenitors, as well as some differentiated lineage cells, are significantly enlarged, while no abnormality was observed in the villi (Figure 6D).

Taken together, deletion of the SMG6’s PIN domain does not cause death in adult mice but specifically compromises tissue homeostasis in the testis and intestine.

## 4. Discussion

Nonsense-mediated mRNA decay is a well-conserved RNA surveillance mechanism. Meanwhile, NMD fine-tunes the abundance of a certain group of gene transcripts [2]. Through these two functions, NMD shapes the cellular transcriptome and safeguards multiple biological processes, including stem cell development and tissue homeostasis [6,39]. Previous studies primarily use the strategy of generating a complete knockout of a specific NMD component in animal models or cell lines and reveal that NMD could serve as a licensing mechanism during cell fate transitions. However, a large body of evidence revealed that the majority of the NMD factors are actively participating in other essential molecular pathways, including DNA damage response, telomere maintenance, and ubiquitination-associated protein degradation, all of which are also plausible for the phenotypes found in NMD factor knockout models [5,6,8,40]. Thus, a more stringent NMD animal model to investigate the biological function of NMD per se is necessary.

SMG6 is the key NMD effector and has been proven to be the only endoribonuclease in the NMD pathway that cleaves the NMD targets near the PTC [25,26,41]. Here, we establish a new NMD-deficient model by specifically deleting SMG6’s endoribonuclease domain (PIN domain), while retaining other parts of the SMG6 protein. Of note, Katsioudi et al. recently reported a NMD loss-of-function mouse line that conditionally expresses a mutant SMG6 protein with the loss of its endoribonuclease activity. They used this mouse model and investigated the NMD function in circadian clock regulation of the mouse liver. In this study, the authors used the PCR assay to investigate the conversion efficacy of the floxed allele to the mutant allele, while the status concerning the mutant *Smg6* mRNA transcript and the expression pattern of the mutated SMG6 protein were not documented [42]. In our study, we extensively investigate the efficacy of the floxed allele (F) conversion into the PIN domain KO allele (∆) and mutant mRNA transcript expression, as well as PIN domain-truncated SMG6 protein detection with antibodies against the C- and N-terminal of SMG6. We showed that the SMG6-PIN^∆^ allele is not an SMG6 null allele, but encodes a truncated SMG6 protein lacking its endoribonuclease domain. These analyses lay a solid foundation in using the *Smg6*-PIN^F/F^ to investigate the *in vivo* functions of NMD per se.

### 4.1. SMG6’s PIN Domain Is Required for NMD In Vitro and In Vivo

The molecular function of SMG6’s PIN domain in NMD has been heavily studied in vitro [23,24,26,43]. In mouse embryonic stem cells, ectopic expression of a truncated SMG6 protein without its PIN domain could not rescue the NMD deficiency in *Smg6* null ESCs, indicating that SMG6’s PIN domain is required for NMD in ESCs [10]. In our analysis, we generated three lines of mESCs expressing truncated SMG6 without its PIN domain from the endogenous *Smg6* mutant locus. We provide several lines of evidence on NMD inhibition in *Smg6*-PIN^Δ/Δ^ ESCs: (1) accumulated RNA transcripts from well-known NMD targets with features of PTC, long 3′UTR, and uORFs; (2) increases in gene-specific PTC^+^ isoforms produced by exon-inclusion and exon-skipping events during alternative splicing. Similar findings of NMD deficiency were found in the tissues of spleen, thymus, kidney, liver, and testis from adult *Smg6*-PIN^Δ/Δ^ mice. Of note, although multiple studies using Western blot analysis revealed that mouse kidneys have the lowest (almost undetectable) expression of NMD components [36,38,44], including SMG6, our data found that the loss of SMG6’s PIN domain still causes NMD inhibition, indicating that the expression level of NMD factors does not correlate with NMD efficiency *in vivo*. Nevertheless, our analyses on mESC lines and multiple tissues from *Smg6*-PIN^iKO^ mice prove that SMG6’s PIN domain (endoribonuclease motif) is required for NMD.

### 4.2. NMD Per Se Is Required for ESC Differentiation and Embryonic Development of Mice

Except for UPF3B [45], which is shown as a weak NMD factor [46], knockout of other individual NMD components causes early embryonic lethality in the mouse [6,13,36]. We previously generated the SMG6 complete knockout mouse line and found that SMG6 null mice die around E7.5-12.5 [10]. Loss of the complete SMG6 protein causes ESC differentiation defects. SMG6 null ESCs expressed a high level of c-MYC protein, and ectopic expression of NMD proficient full-length or N-terminal truncated SMG6 (preserving the NMD function) could efficiently reduce c-MYC expression and rescue the SMG6 null differentiation defects in vitro [10]. Huth et al. used the CRISPR–Cas9 strategy and generated SMG6 null ESCs and found the similar differentiation defects of SMG6 null ESCs. However, under the 2i culture condition to preserve the naïve state of ESC, SMG6 null ESCs express a decreased level of c-MYC [12]. The analyses conducted in these two studies are based on the genetic background of complete SMG6 loss. The current study intends to establish a direct link between SMG6’s endoribonuclease domain in ESC differentiation and embryonic development. *Smg6*-PIN^Δ/Δ^ mice are embryonic lethal within the time window E7.5–13.5, similar to SMG6 complete knockout embryos. Furthermore, the morphology and cell proliferation of *Smg6*-PIN^Δ/Δ^ ESCs are identical to control ESCs. The *Smg6*-PIN^Δ/Δ^ ESCs under feeder-layer culture conditions express higher levels of c-MYC and exhibit differentiation defects in the spontaneous differentiation assay. These data indicate that higher c-MYC expression upon NMD deficiency could be dependent on specific culture condition, i.e., feeder-layer support, since we previously also showed that under feeder-layer support, SMG5 null ESC expressed higher levels of c-MYC, which could be restored by ectopic expression of full-length SMG5 protein [13]. This finding highlights that NMD and its targets are cell type and cell status specific [6,47]. Nevertheless, our data establish a molecular link between NMD per se and cell fate determination of mESCs.

### 4.3. NMD and Tissue Homeostasis

NMD is considered a licensing mechanism for cell fate transitions [6]. This hypothesis has been extensively investigated on the embryonic development and tissue-specific stem cell differentiation using animal models [5,6,39,48]. For example, induced deletion of *Upf2* specifically in hematopoietic stem cells and hematopoietic progenitors in adult mice causes rapid lethality, indicating that UPF2-NMD is essential for the viability of proliferative cells [30]. In another study, Steiner et al. used a whole-body inducible transgenic Cre line (Cre-ER^T2+^), and generated the *Smg7*-inducible knockout mice [44]. They found that induced SMG7 deletion in adult mice did not affect tissue homeostasis in the short term (14 days after the Tamoxifen treatment). In our study, we found that induced SMG6 deletion in adult mice does not trigger death; however, several organs, including the testis and intestine, showed abnormalities upon loss of SMG6’s endoribonuclease domain/NMD activity.

In adult mouse, testis continuously produces mature sperms to maintain the reproductivity. The spermatogenesis process in adult mouse includes the differentiation of spermatogonia into spermatocytes, meiosis of spermatocytes, and the maturation of round spermatids into elongated spermatids, during which cell fate determinations are actively executed [49]. NMD factors are highly expressed in the mouse testis [17,36,38,50]. SMG6 is localized in the chromatoid body (CB) of late spermatocytes and round spermatids [51,52]. The chromatid body is a male germ cell-specific RNA granule, which functions in the transition from meiotic spermatocytes to the post-meiotic spermatids. Complete knockout of SMG6 in differentiating spermatogonia revealed a defect in the transition from round spermatids towards the elongated spermatids [38]. Testes from our *Smg6*-PIN^iKO^ mice, at 1 month after Tamoxifen treatment, showed severe atrophy. The seminiferous tubules of the *Smg6*-PIN^iKO^ testis have a dramatic reduction in elongated spermatids, indicating that NMD per se is required for spermatogenesis, especially during the transition from round spermatids to elongated spermatids.

The intestine is another constantly renewing tissue in the mouse. The somatic cell lineages in the intestinal epithelium originate from the intestinal stem cells (ISCs) residing in the mouse crypt [53]. In contrast to the reduced cellularity in the *Smg6*-PIN^iKO^ testis, loss of SMG6’s PIN domain caused enlarged intestinal crypts. In the intestinal crypts, intestinal stem cells self-renew to maintain the stem cell identity, and differentiate into transient amplifying progenitors (TA cells) and finally into different cell lineages of the villi [54]. Our data suggest that NMD loss may promote the hyperproliferation of ISCs and TA cells. However, the current study could not reach a conclusion that SMG6’s PIN domain regulates the fate transition of ISCs and TA cells in a cell-autonomous manner. The mechanism of NMD in intestinal stem cell fate determination requires more detailed analysis with tissue-specific Cre lines, such as Villin-Cre.

In sum, we conducted a detailed characterization of our newly generated conditional knockout mouse line that could be used to specifically deactivate SMG6’s endoribonuclease domain (PIN domain). Our study discloses that NMD per se is required for mESC differentiation and embryonic development during organogenesis. Furthermore, NMD per se is essential for tissue homeostasis in the adult mouse, where cell fate transitions are ongoing. The current study establishes a causal link between NMD per se and cell fate determination.

## 5. Limitations of the Study

SMG6 is the only endoribonuclease in the nonsense-mediated mRNA decay pathway. Here, we generated a conditional mouse line with the capability of specifically deactivating SMG6-mediated endoribonuclease/NMD activity, but not a complete knockout of SMG6 protein. Although our data demonstrated that deletion of *Smg6*’s exon 18 in mouse embryonic stem cells and in multiple tissues from adult mice resulted in accumulation of classical NMD target transcripts with the features of premature termination codons, long 3′UTRs or 5′ uORFs, some questions are still open for further investigations.

One open question is the dynamics of the SMG6 PIN domain-truncated protein during the NMD process, including its interaction with phosphorylated UPF1 and its ability to recruit PP2A to dephosphorylate p-UPF1. Furthermore, a direct analysis on the telomere maintenance via the telomere-FISH with PNA probes could be necessary to fully characterize SMG6’s endoribonuclease activity in mammalian telomere regulation. RNA-seq analysis of ESC samples established in the current study or of E3.5 blastocysts isolated from the intercross of *Smg6*-PIN^+/∆^ mice could elucidate transcriptomic dysregulation in the absence of SMG6-NMD. Furthermore, RIP-Seq with the SMG6-specific antibodies could reveal direct SMG6-NMD targets in ESCs.

The current study gave a pilot analysis on the *in vivo* functions of SMG6’s PIN domain. Deletion of SMG6’s NMD domain in adult (male) mice compromises homeostasis in testes and intestines in the short term, and warrants further investigation into the mechanisms underlying cell fate determination using tissue- or cell-type-specific Cre lines. The long-term effect on SMG6’s NMD deactivation is another interesting topic to follow, since decreased NMD activity has been correlated with aging [55,56].

## Figures and Tables

**Table 1:** Primers used for qPCR analysis.

Gene	Forward Primer (5′-3′)	Reverse Primer (5′-3′)
*Smg6 (E18)*	AAGGATTACATGCCCACCAG	TCACAGGCACATTCCTTGTC
*Smg5*	GGAACTGCTGTGGAGAAAGG	AGCGACCAGATGAGTCCTGT
*Smg6 (E2)*	CTACCGCACTTGCAGTACCAG	CGACATCCATTATCGGTCAGG
*Smg7*	AACCCAAATCGAAGTGAAGTCC	ACACCGTACACAGTTCCTGTAA
*Atf4*	CACAACATGACCGAGATGAG	CGAAGTCAAACTCTTTCAGATCC
*Eif4a2*	ATAGCGGCAGTTGATGACGA	CCCTCTGCCAATTTCGCTCT
*Hnrnpl*	TCGCAGTGTATGTTTGATGGG	CTGGCGTTTGTTGGGGTTAC
*Snord22*	GCCAGGCCTGTTCAATTTTA	TGCCTGAGATTTGTCACCAG
*Gas5*	TTTCCGGTCCTTCATTCTGA	TCTTCTATTTGAGCCTCCATCCA
*1810032O08Rik*	GCACAAGGGCCTTCAGGAT	TTGGCACTGATGGTCCACTG
*Pdrg1*	GAAAGGCTGCGGAGTCAACTT	GGGCTGAGGGGATTCAGGTT
*Rassf1*	CTGTAGAGCGGGAGACACC	GCACTGAAACAGGACGCACT
*Snhg12*	GGTCCCTGTCTGTTTTCGTT	TCTTCTGGTCTCCCTCCTCA
*Auf1*	GAAAGTATCCAGGCGAGGTG	GGCCTGTGAGAATCGTGAAG
*β-Actin*	AGAGGGAAATCGTGCGTGAC	CAATAGTGATGACCTGGCCGT

**Table 2:** Primers used for RT-PCR on alternative spliced variant transcripts.

Gene	Forward Primer (5′-3′)	Reverse Primer (5′-3′)
*Pkm2*	GCAGCAGCTTTGATAGTTCTC	GCCAAGTTTACACGAAGGTC
*Rps9*	CCAAATCTATTCACCATGCCC	GATGTGCTTCTGAGAGTCCA
*Srsf3*	AAGAGAAGTCGGAATCGTGG	GACCTTTCTCTTCTCCTATCTC
*Eif4a2*	GGATTGACGTGCAACAAGTG	TAGGTCAGCCACATTCATGG
*Jmjd6*	GTTGTCCTCAACCTTGACAC	CTAGAGGAGCTAGAAGAGTCG
*Hnrnph1*	GGTCCAAATAGTCCTGACAC	CTCTGCCAATGCTGTTATACC
*Ptbp2*	TCTCAGTCCTTTGGCTATTCC	CATCAGCCATCTGTATCAGAG
*Ccar1*	GGAATGAAAGGCAAGGATGA	GGGTAGGAGTGGCGATCTCT
*Nfyb*	CCTCCCAGCTAGGGATTTCT	GTCTTCCGCTTCTCCTGATG
*Sf1*	TGGAACCAAGACACAATGGA	GGGTTGAGAGCAACCATCTC

## Data Availability

The original contributions presented in this study are included in the article/Supplementary Materials. Further inquiries can be directed to the corresponding author.

## Supplementary Materials

The following supporting information can be downloaded at https://www.mdpi.com/article/10.3390/cells15030282/s1. Figure S1: Comparison of wild-type and PIN domain deleted SMG6 protein; Figure S2: Schemes to generate the mouse lines and ESC lines used in this study; Figure S3: RT-PCR analysis to show the accumulation of PTC^+^ transcripts produced by the exon-inclusion and exon-skipping events of alternative splicing in kidney, spleen, liver, and testis of Tamoxifen-treated *Smg6*-PINF/F Cre-ER^T2−^ (Control) and *Smg6*-PINF/F Cre-ER^T2+^ (*Smg6*-PINiKO) mice.
