# Supplementary material for: SMG6’s PIN (PilT N-Terminus) Domain Is Required for Nonsense-Mediated mRNA Decay (NMD) In Vivo
Source: Cells. 2026 Feb 2;15(3):282. doi: 10.3390/cells15030282 (PMC12896539; doi:10.3390/cells15030282)
Supplement: Supplementary file 1 [file cells-15-00282-s001.zip › cells-4098060-supplementary.pdf]

>mouse SMG6-Full length (WT), 1418 AA, Predicted molecular weight 160.5 KD

MAEGLERVRISASELRGILATLAPQAGSRENMKELKEPRQRKDNRRPDLEIYKPGLSRLRNRPKTKEASGNEEFKDEIVND
RDSSAVGNDTQLIQVCKELDSQQQNGPIDAENSQAQETFPKTVGLEDRSLKIIKRSKKPDLQIYQPGRRLQTITKESAGRA
DEEEILNQVEQLRIEEDECKGEAIKEEVNNKPDKTEIEKHQSNDRVRTAKGEKGKKIEKGEGSKKVADDSVPGKPGSVKRY
SRSDKRRNRYRTCSTSSAGSNNSAEGAGLTDNGCRRRRQDRAKERPRLKKQVSLSSTDSLDEDRVDEPDVLGSRRSSE
RKKHLERNWSGCGEGEQKSNGKENRSALRVTFDAETMSKDSPVVRSVKDNVDRMKSDKGPSSGGKGSEKQELRHPRQ
ELRDRGRGILILPAHTALSVSSSGSPESTPLGPRLLFGSGSKGSRSWGRGGTTRRLWDPNNPDQKPALKSQTPQLHFLDT
DDEISPTSWGDSRQAQASYYKFQNSDNPYYYPRTPGPASQYPYAGYSPLQYPVGPTNGMYPGAYYPGYPAPSGQYVC
SPLPASTMSPEEIEQHVRNMQQQELHRLLRVADNQELQLSNLLSRDRISTEGMEKMAQLRTELLQLYERCILLDIEFSDSQ
NVDQILWKNAFYQVIEKFRQLLKDPNSENPEQIRNRLLELLDEGSDFFDSLLQKLQVTYKFKLEDYMDGLAIRSKPLRKTVK
YALISAQRSMICQGDISRYREQANDTANYGKARSWYLKAQHIAPKNGRPYNQLALLAVYTRRKLDAVYYYMRSLAASNPIL
TAKESLMSLFEETKRKAEQMEKKQHEEFDMSPDKWRKGKKSTFRHVGDDTTRLEIWIHPSHSRSAQGTESGKDSEQEN
GLGSLSPSDLNKRFILSFLHAHGKLFTRIGMETFPAVAEKVLKEFQVLLQHSPSPIGSTRMLQLMTINMFAVHNSQLKDCFS
EECRSVIQEQAASLGLAMFSLLVQRCTCLLKDSAKAQLSSPEDQEDQDDIKVSSFVPDLKELLPSVKVWSDWMLGYPDT
WNPPPTSLDLPLQVAVDVWSTLADFCNILTAVNQSEVPLYKDPDDDLTLLILEEDRLLSGFVPLLAAPQDPCYVEKTSDKVI
AADCKRVTVLKYFLEALCGQEEPLLAFKGGKYVSVAPVPDTMGKEMGSQEGKQLEDEEEDVVIEDFEEDSEAEGSGGED
DIRELRAKKLALARKIAEQQRRQEKIQAVLEDQSQMRQMELEIRPLFLVPDTNGFIDHLASLARLLESRKYILVVPLIVINELD
GLAKGQETDHRAGGYARVVQEKARKSIEFLERRFESRDSCLRALTSRGNELESIAFRSEDITGQLGNNDDLILSCCLHYCK
DKAKDYMPTSKEEPIRLLREVVLLTDDRNLRVKALTRNVPVRDIPAFLTWAQVG*

D1352 Encoded by Exon 18

D1391

>mouse SMG6-PIN△, 1360 AA, Predicted molecular weight 153.9KD

MAEGLERVRISASELRGILATLAPQAGSRENMKELKEPRQRKDNRRPDLEIYKPGLSRLRNRPKTKEASGNEEFKDEIVND
RDSSAVGNDTQLIQVCKELDSQQQNGPIDAENSQAQETFPKTVGLEDRSLKIIKRSKKPDLQIYQPGRRLQTITKESAGRA
DEEEILNQVEQLRIEEDECKGEAIKEEVNNKPDKTEIEKHQSNDRVRTAKGEKGKKIEKGEGSKKVADDSVPGKPGSVKRY
SRSDKRRNRYRTCSTSSAGSNNSAEGAGLTDNGCRRRRQDRAKERPRLKKQVSLSSTDSLDEDRVDEPDVLGSRRSSE
RKKHLERNWSGCGEGEQKSNGKENRSALRVTFDAETMSKDSPVVRSVKDNVDRMKSDKGPSSGGKGSEKQELRHPRQ
ELRDRGRGILILPAHTALSVSSSGSPESTPLGPRLLFGSGSKGSRSWGRGGTTRRLWDPNNPDQKPALKSQTPQLHFLDT
DDEISPTSWGDSRQAQASYYKFQNSDNPYYYPRTPGPASQYPYAGYSPLQYPVGPTNGMYPGAYYPGYPAPSGQYVC
SPLPASTMSPEEIEQHVRNMQQQELHRLLRVADNQELQLSNLLSRDRISTEGMEKMAQLRTELLQLYERCILLDIEFSDSQ
NVDQILWKNAFYQVIEKFRQLLKDPNSENPEQIRNRLLELLDEGSDFFDSLLQKLQVTYKFKLEDYMDGLAIRSKPLRKTVK
YALISAQRSMICQGDISRYREQANDTANYGKARSWYLKAQHIAPKNGRPYNQLALLAVYTRRKLDAVYYYMRSLAASNPIL
TAKESLMSLFEETKRKAEQMEKKQHEEFDMSPDKWRKGKKSTFRHVGDDTTRLEIWIHPSHSRSAQGTESGKDSEQEN
GLGSLSPSDLNKRFILSFLHAHGKLFTRIGMETFPAVAEKVLKEFQVLLQHSPSPIGSTRMLQLMTINMFAVHNSQLKDCFS
EECRSVIQEQAASLGLAMFSLLVQRCTCLLKDSAKAQLSSPEDQEDQDDIKVSSFVPDLKELLPSVKVWSDWMLGYPDT
WNPPPTSLDLPLQVAVDVWSTLADFCNILTAVNQSEVPLYKDPDDDLTLLILEEDRLLSGFVPLLAAPQDPCYVEKTSDKVI
AADCKRVTVLKYFLEALCGQEEPLLAFKGGKYVSVAPVPDTMGKEMGSQEGKQLEDEEEDVVIEDFEEDSEAEGSGGED
DIRELRAKKLALARKIAEQQRRQEKIQAVLEDQSQMRQMELEIRPLFLVPDTNGFIDHLASLARLLESRKYILVVPLIVINELD
GLAKGQETDHRAGGYARVVQEKARKSIEFLERRFESRDSCLRALTSRGNELESIAFRSEDITGQLRSQSACYGRWCC*

Molecular weight (MW) of protein prediction website: https://www.sciencegateway.org/tools/proteinmw.htm

Figure S1. Comparison of wild-type and PIN domain deleted SMG6 protein. Note: Deletion of *Smg6* exon 18 will cause the removal of last 70 amino acids in SMG6's C-terminal.

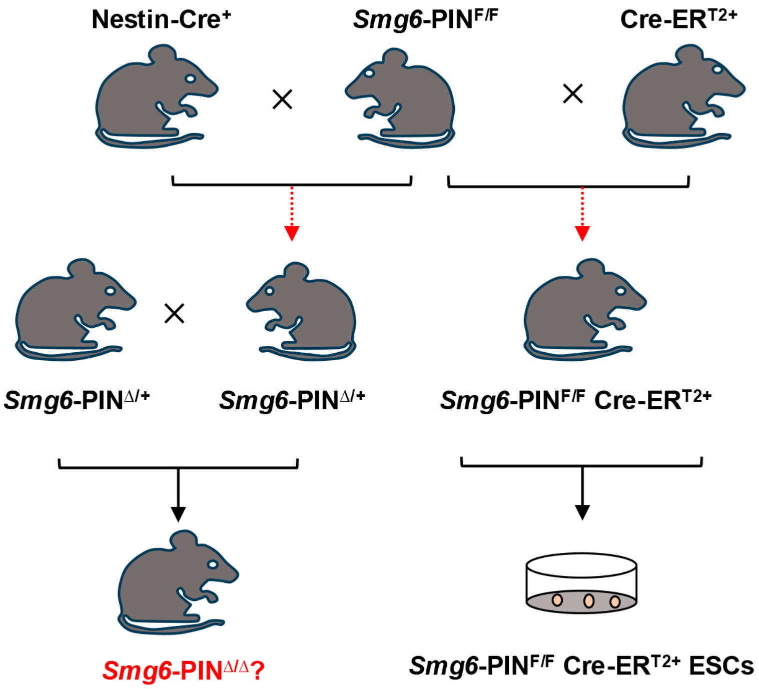

Figure S2. Schemes to generate the mouse lines and ESC lines used in this study.

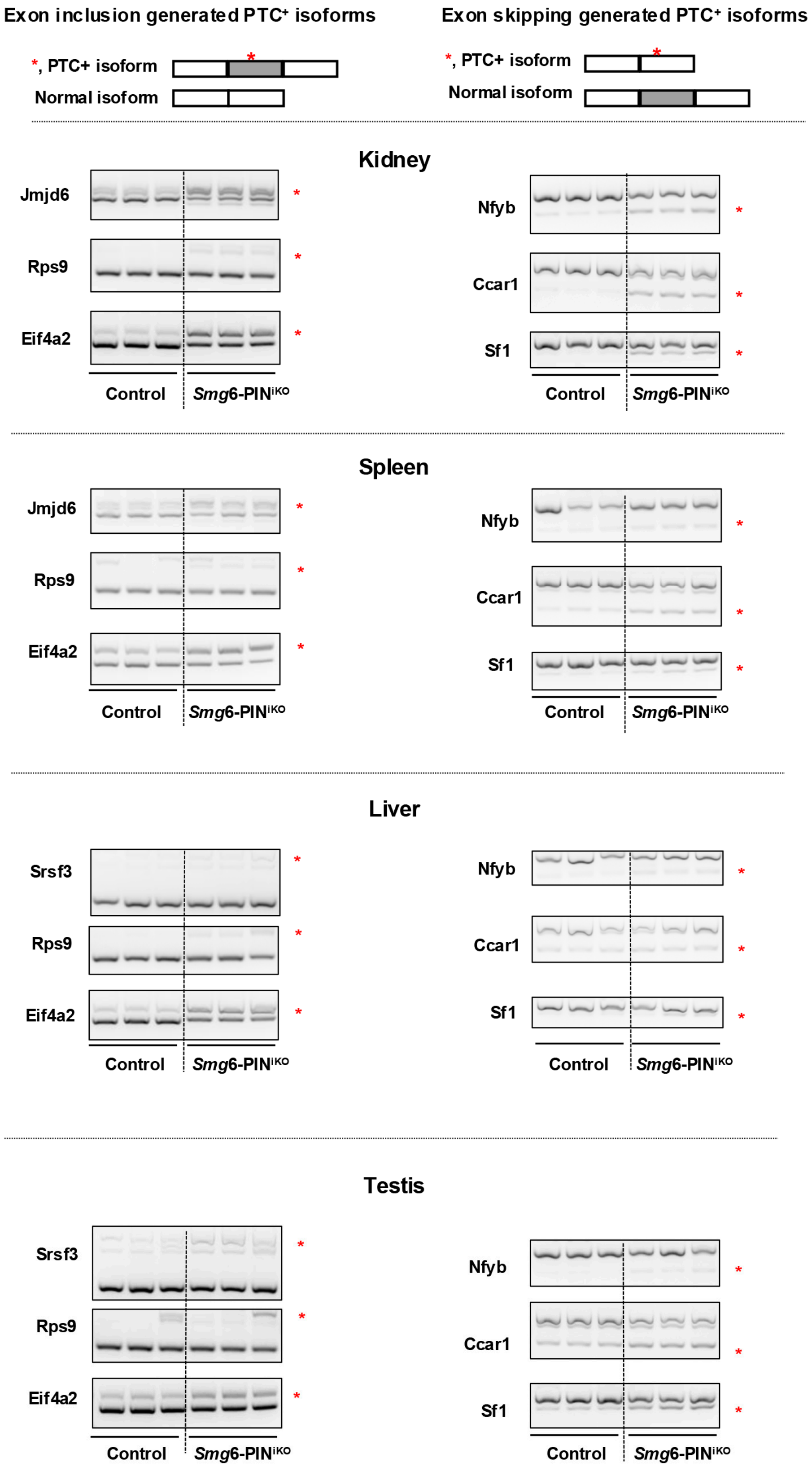

Figure S3. RT-PCR analysis to show the accumulation of PTC$^+$ transcripts produced by the exon inclusion and exon skipping events of alternative splicing in kidney, spleen, liver, and testis of Tamoxifen treated *Smg6*-PIN$^{F/F}$ Cre-ER^{T2-} (Control) and *Smg6*-PIN$^{F/F}$ Cre-ER^{T2+}(*Smg6*-PIN^{iKO}) mice. Red asterisks denote the PTC$^+$ isoforms. RNA samples are collected from mice one month after the last Tamoxifen injection.
